# Young Female With Hypereosinophilia, Rash, and Gait Disturbance: A Case Report

**DOI:** 10.7759/cureus.56498

**Published:** 2024-03-19

**Authors:** Praveen Hariharan, Lina Nemchenok, Mohanad Hadi, Vaugh Williams, Angela Caliendo

**Affiliations:** 1 Emergency, Brown University, Providence, USA; 2 Medicine, Brown University, Providence, USA; 3 Rheumatology, Brown University, Providence, USA; 4 Infectious Disease, Brown University, Providence, USA

**Keywords:** churg-strauss syndrome, eosinophilic asthma, disease relapse, mononeuritis, rash, hypereosinophilia

## Abstract

Eosinophilia is known to be associated with a multitude of co-morbidities. However, unexplained eosinophilia poses a diagnostic challenge, and the methods used to investigate unexplained eosinophilia vary from region to region. In this case report, we describe a unique case of a young female presenting with marked eosinophilia to a tertiary hospital in the northeastern United States. Our patient presented with a few weeks of lower extremity rash, gait instability, and new onset marked eosinophilia. We further report the investigations undertaken during the hospitalization to highlight the broad differential diagnoses. Later, we provide a consolidated diagnosis of eosinophilic granulomatosis with polyangiitis (EPGA) based on the clinical context. Our patient was eventually started on a high-dose steroid taper. In the following weeks, while we noted gait improvement, we observed biomarker (eosinophilia) relapse after steroid taper. Depending on symptom progression, we planned for future remission induction with immunomodulatory agents. The report further discusses the pleomorphic presentation of EPGA cases, the natural course of disease, and currently available prognostic indices.

## Introduction

We can broadly classify eosinophilia as reactive (non-clonal), clonal dysplastic disorders, and idiopathic hypereosinophilic syndromes [1,2]. Clinical manifestations of patients presenting with eosinophilia often reflect the underlying etiology. For instance, the etiology of reactive eosinophilia could range from infections, allergic drug reactions, autoimmune, or solid or hematologic tumor-related conditions. Clonal disorders include a range of myeloproliferative disorders, whereas idiopathic syndromes include presentations with no ascertainable cause [1,2]. Eosinophilia can be transient, persistent, or exhibit a waxing and waning pattern. Peripheral blood eosinophilia recorded on at least two occasions at least four weeks apart is considered persistent eosinophilia [3]. In addition, persistent eosinophilia can cause tissue infiltration, leading to end-organ damage. Eosinophilic granulomatosis with polyangiitis (EPGA) is an example of persistent eosinophilia associated with multiple end-organ effects ranging from cutaneous, cardiopulmonary, neurologic, renal, and gastrointestinal manifestations [4].

## Case presentation

**Figure 1:**
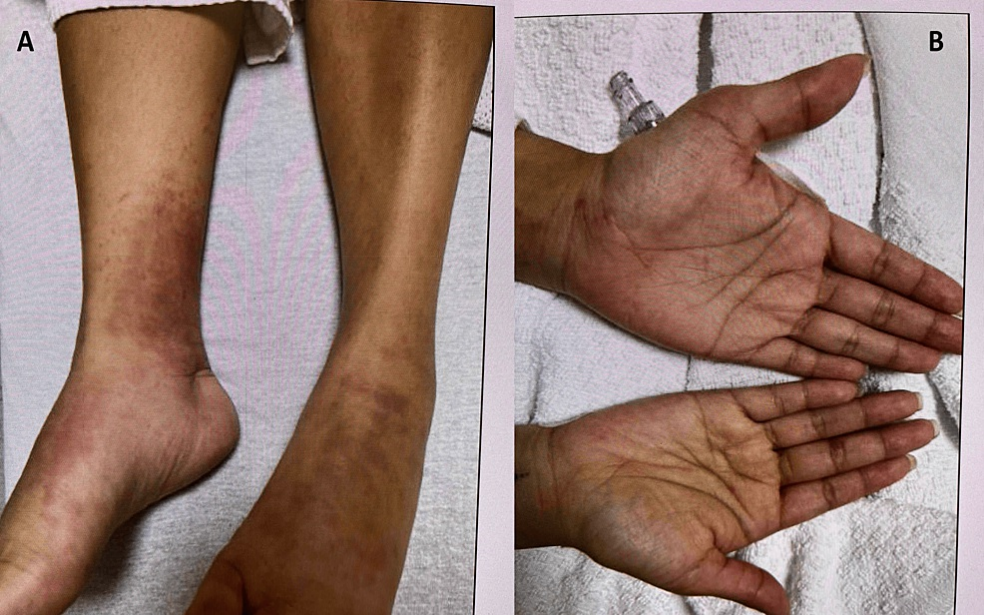
(Day 0) Skin lesions of the lower extremities (A) and hands (B)

We describe a case of a 36-year-old female with a history of mild persistent asthma who presented to the emergency department (ED) with new onset maculopapular rash on the upper and lower extremities, progressive gait instability, polyarthralgia, lower extremity pain, and paresthesia for two weeks. The patient initially reported to urgent care with leg pain about ten days before the ED presentation and was issued gabapentin for presumed sciatica. A week later presented to a dermatology clinic with a bilateral lower extremity maculopapular rash (Figures 1A, 1B) and was issued topical steroids. After a day she came to the ED with progressive gait difficulties, rash, and lower extremity paresthesia. She declined any recent travel, new medications (other than the gabapentin), and substance use. Other medical history included severe food allergies (seafood, soy, and spinach), a prior cesarean section, cholecystectomy, and appendectomy.

On presentation, she had a temperature of 36.5°C, breathing at 16 breaths per minute with a blood pressure of 137/89 mmHg, and pulse of 86 beats per minute, saturating at 99% on room air. Upon examination, we noted no wheezing, and a regular heart rate with a faint systolic murmur at the left lower sternal border. She had no abdominal organomegaly. On a neurologic exam, she was alert, with decreased vibration and touch on the dorsal and plantar feet bilateral, 3/5 plantar/dorsiflexion, preserved patellar reflexes with absent ankle jerks, and an unremarkable cerebellar exam. A dermatologic exam revealed maculopapular rash predominantly in the distal lower extremities (Figures 1A-1C) but also noted on the palms.

Baseline labs revealed a white cell count (WBC) of 29,000 per microliter (μL) (no blasts) with marked eosinophilia at 66% (absolute count-20,7000 per μL), hemoglobin of 13.2 grams per deciliter, and platelets at 290,000 per μL. Electrolytes, hepatic function panel, and a urinalysis were normal. The sedimentation rate was 37 mm/hr (average <20.0 mm/hr), and C-reactive protein was 14.9 mg/L (normal <10.0 mg/L). She had a detailed radiographic assessment, including a chest-abdomen-pelvis CT and MR lumbar spine with and without contrast, without pathologic abnormalities. A fiberoptic nasolaryngoscopy revealed typical anatomic landmarks and no nasal polyps.

The immunology panel revealed elevated immunoglobulin (Ig) E levels of 5,328 IU/mL (normal <209 IU/mL) and IgG4 levels of 328 mg/dL (normal <123 mg/dL). IgA, IgM, cryoglobulins, and tryptase levels were within normal limits. The autoimmune panel revealed negative antinuclear antibodies, antineutrophilic cytoplasmic antibodies (ANCA), anti-cyclic citrullinated peptide, Sjogren panel, antistreptolysin panel, anti-myeloperoxidase, and antiproteinase 3 antibodies. Rheumatoid factor was 251 IU/mL (normal <30 IU/mL) with normal complement levels. The hematologic malignancy assessment was negative including, serum and urine electrophoresis, peripheral blood flow cytometry, fluorescence in situ hybridization for any evidence of 4q12 (CHIC2) deletion, and polymerase chain reaction test for KIT (D816V) point mutation, BCR-ABL1 fusion transcripts, and JAK2 pV617F mutation. For infectious workup, a peripheral smear, PCR screen for Anaplasma, Ehrlichia, COVID-19, Influenza, RSV, and antibody screen for Lyme, Syphilis, and HIV were negative. In addition, she had a negative acute hepatitis panel and was seronegative for Histoplasma, Coccidiodes spp, Blastomyces dermatitidis, Parvovirus, Bartonella spp, Rickettsia spp, Stronglyoides, and Trichinella. She had a negative TB interferon-gamma release assay. Stool studies did not reveal any parasites.

**Figure 2:**
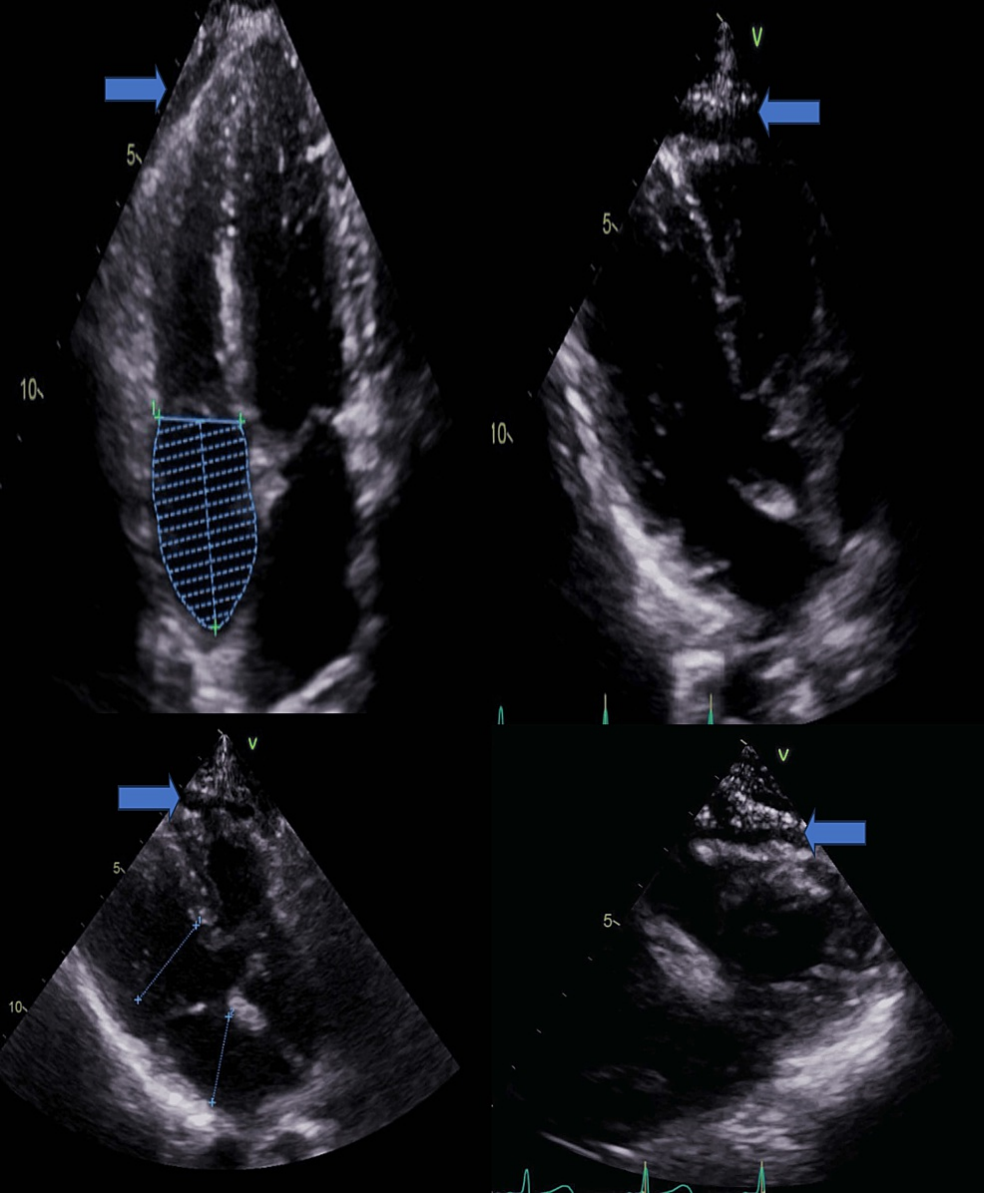
Trace pericardial effusion arrow marked

We noted distal limb length-dependent sensorimotor axonal neuropathy in nerve conduction studies. An echocardiogram showed a small pericardial effusion (Figure 2) with trace tricuspid regurgitation.

**Figure 3:**
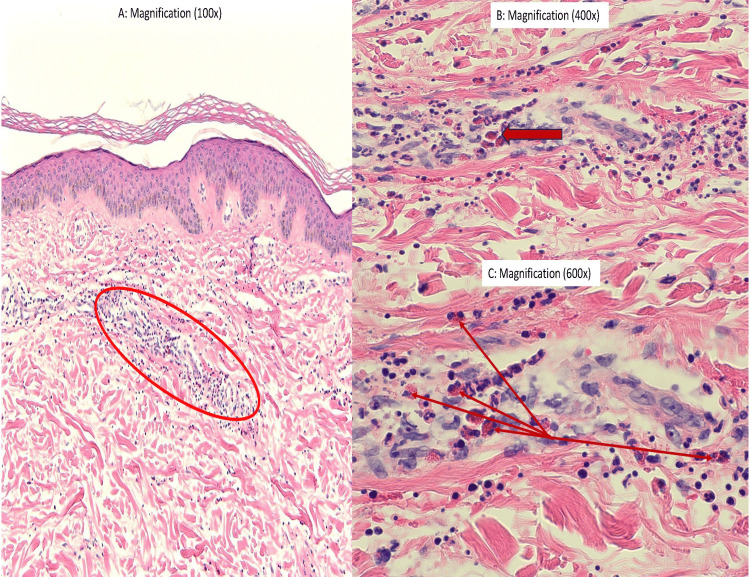
H&E stain of right leg skin punch biopsy (A) Neutrophilic karyorrhexis (red circle) affecting small vessels. (B, C) Arrow-marked extravascular eosinophils.

A punch skin biopsy of the right leg rash revealed leukocytoclastic small vessel vasculitis with increased eosinophils (Figures 3A-3C). Conspicuous granulomata were absent in the observed sections. Immunofluorescent staining demonstrated focal vascular C3, IgM 1+, minimal IgA, perivascular fibrinogen, and no IgG. The immunofluorescent staining helped rule out certain autoimmune conditions like dermatitis herpetiformis, IgA disease, and pemphigoid lesions. We further ruled out urticarial vasculitis associated with certain autoimmune conditions (Lupus or Sjogren) and infectious conditions like Hepatitis B/C based on serologic testing. While the appearance of distal extremity rash raised concern for Kaposi sarcoma (KS), the relatively acute onset, lack of immunosuppression, lack of high-risk sexual behavior, and negative HIV serologies made KS less likely.

Her constellation of symptoms with known asthma, new onset eosinophilia, peripheral neuropathy, pericardial effusion, and a skin biopsy demonstrating eosinophilic vasculitis, was highly suggestive of EGPA aka Churg-Strauss syndrome (CSS) [2,5].

**Figure 4:**
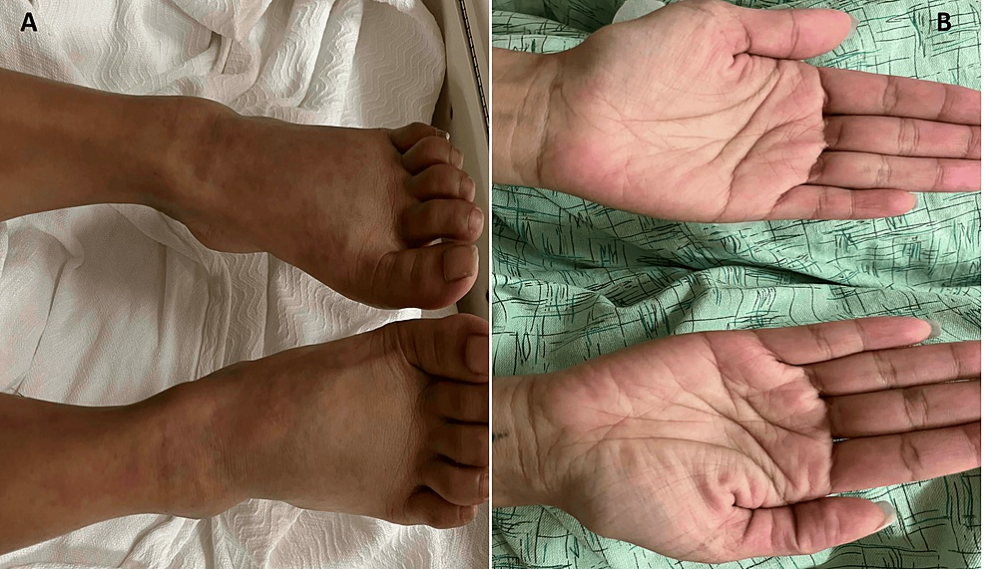
(Day 7) Skin lesions of the lower extremities (A) and hands (B)

During her hospital course, the rash started to recede (Figures 4A, 4B). On day 7, her eosinophil count peaked at 30,000 per μL with a WBC of 41,600 per μL. Hence, she was started on a three-week prednisone taper. After two weeks of therapy, her repeat complete blood count revealed a WBC of 12,200 per μL with eosinophils of 4,300 per μL. Further review of her historical blood counts revealed mild eosinophilia of 1,900 cells/L with a WBC of 11,300 per μL during an office visit about two years before her recent presentation.

At two weeks follow-up, she had positive antibody titers for Toxocara species. The patient neither had any clinical manifestations of active Toxocariasis like pneumonitis, hepatosplenomegaly, fever, or ocular manifestations nor radiographic lesions consistent with Toxocara [6]. Moreover, Toxocara's seroprevalence has been estimated at up to 5% in the US population [7]. While an empiric five-day course of albendazole was prescribed out of an abundance of caution, it was believed the seropositivity was likely cross-reactivity or distant exposure as the patient migrated from Thailand at the age of two.

Her mobility improved in one month with outpatient physical therapy. Given symptom improvement, she initially declined a sural nerve biopsy. However, after two months of observation therapy, she had persistent feet paresthesia; her eosinophilia relapsed with WBC at 22,300 per μL, including eosinophils of 14,000 per μL and now she is agreeable to confirmatory biopsy and further immunosuppressive therapy.

## Discussion

EPGA is a systemic disease with pleiotropic symptoms, and its manifestations can be broadly categorized into two subsets: vasculitic and eosinophilic [4]. In addition to peripheral eosinophilia, the eosinophilic manifestations involve pathologies related to the heart, lung, and gastrointestinal tract. The vasculitic manifestations involve peripheral neuropathy, glomerulonephritis, and skin lesions. CSS as a disease entity was initially described in 1951, and since then, the definition of the entity has evolved [5,8,9]. In 1949, Jacob Churg and Lotte Strass reported a series of autopsies to investigate the relationship between asthma and periarteritis nodosa-like vascular lesions [8]. Based on their observations, CSS was defined by asthma, fever, eosinophilia, extravascular and vascular granuloma aka “allergic granuloma” [8]. Subsequently, EPGA has been considered as one of the manifestations of systemic necrotizing vasculitides [10]. More recently, given the heterogeneity of EPGA presentations, surrogate manifestations of eosinophilic vasculitis like mononeuritis multiplex, pericarditis, myocarditis, and hematuria, in addition to peripheral eosinophilia, are being considered under EPGA [9]. ANCA serologies have been used as surrogates of active vasculitis as an alternative to organ biopsy-proven vasculitis [9].

While the lack of sural nerve biopsy results impedes us from providing a gold standard tissue diagnosis of EPGA, many clinicopathologic findings raise our suspicion for EPGA [11]. In terms of clinical symptoms, our patient had a history of asthma, peripheral neuropathy, arthralgias, and mild pericardial effusion [11]. Regarding immunohematologic findings, she had marked eosinophilia, elevated IgG4 levels, and IgE levels consistent with those reported in active EPGA [4,12]. While the skin biopsy did not report granulomatous findings possibly related to the early phase of the disease course, it did demonstrate leukocytoclastic vasculitis and eosinophil infiltration compatible with EPGA [4,5,13]. Moreover, certain EPGA cases lack characteristic pathologic findings yet follow similar clinical patterns [9,14]. We can also classify EPGA based on ANCA positivity. In one study, when compared to ANCA-negative cases, ANCA-positive tended to manifest more renal disease, arthralgia, mononeuritis multiplex, and less myocarditis [9]. Our case had negative ANCA, and neither had glomerulonephritis or myocarditis but had arthralgia and peripheral neuropathy on presentation. Her remote mild eosinophilia (>1,500 per μL) about two years ago raised concerns about idiopathic hypereosinophilic syndrome [15]. However, we confirmed this result as an outlier when we traced and compared her blood counts in the preceding decade.

EPGA treatment varies according to the severity of manifestations. Systemic glucocorticoids remain the mainstay treatment for EPGA [16]. For severe cases, the American College of Rheumatology/Vasculitis Foundation recommends pulse cyclophosphamide or rituximab for remission induction. ANCA serologies have also been used to determine the agent of choice in severe cases [16]. In non-severe cases, in addition to glucocorticoids, anti-IL-5 agents like mepolizumab have been recommended for remission induction [16].

EPGA, in general, has a favorable prognosis, and a prognostic index called the revised 5-factor score (FFS) has been described to identify high-risk groups [10]. Revised FFS criteria include the presence of one or more of the following: age > 65 years, elevated serum creatinine (>150 μmol/L), gastrointestinal tract involvement, cardiomyopathy, and absence ear-nose throat manifestation [10]. Accordingly, the five-year mortality of FFS 0, 1, and >=2 were 9%, 21%, and 40%, respectively. Our case had an FFS of 1 (based on the absence of ear-nose-throat manifestation) on presentation and is expected to have a somewhat unfavorable prognosis. However, sudden cardiac death from myocarditis remains a rare frightening complication for patients diagnosed with EPGA [13].

## Conclusions

In summary, eosinophilia in the setting of other systemic complaints warrants thorough investigation. EPGA is a relatively rare clinical entity and poses a diagnostic challenge. Our case highlights one of the manifold manifestations of EPGA and the necessity of continued vigilance to address the waxing and waning clinical course. Systemic immunosuppressive therapy remains the mainstay treatment for achieving clinical remission.
